# Epidemiology of Peripheral Vascular Disease in the Long Life Family Study (LLFS)

**DOI:** 10.1093/geroni/igab046.209

**Published:** 2021-12-17

**Authors:** Allison Kuipers, Ryan Cvejkus, Emma Barinas-Mitchell, Mary Feitosa, Joanne Murabito, Joseph Zmuda

**Affiliations:** 1 University of Pittsburgh, Pittsburgh, Pennsylvania, United States; 2 Washington University School of Medicine in St. Louis, St. Louis, Missouri, United States; 3 Boston University, Framingham, Massachusetts, United States

## Abstract

Atherosclerotic occlusion of peripheral arteries is a major contributor to morbidity and mortality in older adults. Our aim was to describe the epidemiology of peripheral artery disease (PAD) and other peripheral vascular disease (OPD) in the LLFS. 3248 individuals from 509 families (1182 probands, mean age 89; 2066 offspring, mean age 60) had doppler ankle-brachial index (ABI) assessment. Measures were performed twice for each posterior tibial artery and minimum of the mean ABI was used. PAD was defined as any ABI<0.9. OPD was defined as any ABI >1.4 or ≥1 non-compressible artery. Stepwise linear or logistic regression determined significant independent clinical and demographic predictors (P<0.05) after adjustment for age, sex, study center, and familial relatedness. Overall, ABI had a median of 1.2 with 7.4% PAD (18.1% probands, 1.2% offspring; P<0.001). OPD prevalence was 10.6% and was more common than PAD in offspring (8.1%). Age-adjusted OPD was higher in men (13.3%) than women (8.3%, P<0.001), while age-adjusted PAD did not did not differ by sex (P=0.45). Predictors of PAD included greater age and systolic blood pressure, lower diastolic blood pressure, prevalent kidney disease, antihypertensive use, and current smoking. Predictors of OPD included greater age, male sex, and current smoking. In these exceptionally long-lived families, PAD was low compared to other epidemiologic studies. However, OPD including non-compressible arteries, a marker of arterial stiffness, was more prevalent than PAD. These findings in long-lived families highlight a need for more epidemiologic research in other peripheral vascular disease in adults from the general population.

